# Provider perceptions of young people living with HIV and unhealthy alcohol use in Southwestern Uganda: a qualitative study

**DOI:** 10.1186/s13722-024-00495-1

**Published:** 2024-09-02

**Authors:** Raymond Felix Odokonyero, Noeline Nakasujja, Andrew Turiho, Naomi Sanyu, Winnie R. Muyindike, Denis Nansera, Fred Semitala, Moses R. Kamya, Anne R. Katahoire, Judith A. Hahn, Carol C. Camlin, Wilson W. Muhwezi

**Affiliations:** 1 Department of Psychiatry, School of Medicine, Makerere University College of Health Sciences, Kampala, Uganda; 2 Mbarara University of Science and Technology, Mbarara, Uganda; 3 Mbarara Regional Referral Hospital, Mbarara, Uganda; 4 Department of Medicine, School of Medicine, Makerere University College of Health Sciences, Kampala, Uganda; 5 Department of Child Health and Child Development, School of Medicine, Makerere University College of Health Sciences, Kampala, Uganda; 6 Division of HIV, ID, and Global Medicine, Department of Medicine, University of California, San Francisco, CA USA; 7 Bixby Center for Global Reproductive Health, Department of Obstetrics, Gynecology & Reproductive Sciences, University of California, San Francisco, CA USA; 8 Center for AIDS Prevention Studies, University of California, San Francisco, CA USA

**Keywords:** Alcohol, HIV/AIDS, HIV care providers, Young people, Uganda, Caring

## Abstract

**Background:**

Unhealthy alcohol use is a common public health problem in HIV care settings in Africa and it affects the HIV continuum of care. In Uganda and other low-income countries, HIV care providers are a key resource in caring for young people (15–24 years) living with HIV (YPLH) with unhealthy alcohol use. Caring for YPLH largely depends on care providers’ perceptions of the problem. However, data that explores HIV care providers’ perceptions about caring for YPLH with unhealthy drinking are lacking in Uganda. We sought to describe the perceptions of HIV care providers regarding caring for YPLH with unhealthy drinking in the Immune Suppression Syndrome (ISS) Clinic of Mbarara Regional Referral Hospital in southwestern Uganda.

**Methods:**

We used semi-structured in-depth interviews (IDIs) to qualitatively explore HIV care providers’ perceptions regarding caring for YPLH with unhealthy alcohol use. The study was conducted at the adolescent immunosuppression (ISS) clinic of Mbarara Regional Referral Hospital. Interviews were tape-recorded and transcribed verbatim. Using thematic content analysis, data from 10 interviews were analyzed.

**Results:**

HIV care providers were concerned and intended to care for YPLH with unhealthy alcohol use. They understood that unhealthy drinking negatively impacts HIV care outcomes and used counseling, peer support, and referrals to routinely intervene. They however, did not apply other known interventions such as health education, medications and follow-up visits because these required family and institutional support which was largely lacking. Additional barriers that HCPs faced in caring for YPLH included; gaps in knowledge and skills required to address alcohol use in young patients, heavy workloads that hindered the provision of psychosocial interventions, late payment of and low remunerations, lack of improvement in some YPLH, and inadequate support from both their families and hospital management.

**Conclusion:**

HIV care providers are important stakeholders in the identification and care of YPLH with unhealthy alcohol use in Southwestern Uganda. There is a need to train and skill HCPs in unhealthy alcohol use care. Such training ought to target the attitudes, subjective norms, and perceived control of the providers.

**Supplementary Information:**

The online version contains supplementary material available at 10.1186/s13722-024-00495-1.

## Background

Alcohol use among people with HIV (PWH) is a public health issue globally [1–3] and is associated with poor outcomes throughout the HIV care continuum. For instance, unhealthy alcohol use, which is characterized by heavy episodic (binge) drinking, at-risk drinking or meeting the criteria for alcohol use disorder (AUD) (Williams et al. 2016), negatively affects people with HIV through increased risk of HIV forward transmission [4], poor retention in care [5], poor adherence to antiretroviral therapy (ART) [6], increased HIV-risk behaviors [7, 8], and increased risk of non-suppression of HIV [9]. Conversely, among PWH, treatment of alcohol use problems is associated with some positive effects such as improved ART adherence in men, fewer readmissions to the emergency department [10, 11], improved acceptance of primary care, decreased hospitalizations, and declines in hospital-related costs [12, 13]. Intervening on unhealthy drinking and treating related alcohol use disorder, therefore, has the potential to positively affect HIV treatment outcomes and reduce HIV transmission [14].

Holding brief discussions with clients in primary healthcare settings by healthcare providers translated into clients’ reducing their subsequent alcohol abuse in studies in Uganda and Zimbabwe [15, 16]. However, many at-risk patients do not receive such provider-delivered discussions, interventions or counselling. Only 11–39% of unhealthy drinkers in sub-Saharan Africa reported receiving any form of intervention during their visit to the health care providers or that their primary care providers discussed concerns regarding alcohol use [17]. Although only a minority of patients in primary care settings reported receiving any form of provider intervention, about 81% of them reported that such interventions and discussions about alcohol issues would be useful [18].

Within primary HIV care settings, the HIV care provider (HCP) is well placed to deal with issues of unhealthy alcohol consumption especially among young people living with HIV (YPLH), since they are clinicians, peer supporters and gatekeepers [19]. However, HCPs are unaware of the burden of drinking by YPLH, and where they are aware of the burden, they lack the knowledge of what to do about it [20]. The fact that many providers are cautious or uncomfortable discussing alcohol use issues with patients is a major barrier to provider discussions of alcohol use problems [20, 21]. Care providers have been found to not only feel uncomfortable discussing alcohol issues, but also to avoid direct discussions even when patients raise the subject, and they give advice that is vague or tenuous [22]. In addition, care providers’ own alcohol use is a barrier which makes them less likely to screen for unhealthy alcohol use among their clients and/or to provide counseling for it [23].

In Southwestern Uganda, alcohol use is prevalent among both young and older people with HIV [24] and specialized alcohol-specific services are lacking. HCPs could pragmatically identify and briefly intervene in unhealthy drinking especially by YPLH. However, little is known about HIV care providers’ experiences and perceptions regarding caring for young people’s alcohol use within the context of HIV. We explored provider perceptions regarding caring for young people living with HIV with unhealthy drinking in a regional referral hospital in Southwestern Uganda.

## Methods

### Study design, participants, setting, and data collection

We conducted an interpretive phenomenological qualitative study to explore the meanings and significance of HIV care providers’ perceptions regarding caring for young people (15–24 years) living with HIV (YPLH) with unhealthy alcohol use, and attending care at the adolescent clinic (also referred to as the youth clinic) of the immunosuppression syndrome (ISS) Clinic of Mbarara Regional Referral Hospital (MRRH). The clinic is staffed by 2 medical doctors, 3 clinicians, 2 nurses, 5 peer counselors, 1 pharmacy staff, 3 laboratory technicians, and 1 data entry staff. Of the 17 staff members working at the adolescent clinic at the time of the study, we conveniently sampled and interviewed 10 providers who met the study inclusion criteria. We conducted the study between June and August 2022 and included HIV care providers who; reported experience in caring for YPLH who drink alcohol, were aged 18 years and above, and consented to the interview. We ensured maximum variation in our sample of providers. The sample thus varied by age (age range of 18 to 60), gender, and professional qualification.

A female research assistant who had conducted other studies at this clinic in the past but not involving care providers, screened and identified potential participants by determining whether the staff member engaged with YPLH in a clinical capacity. Clinical capacity was defined as being involved in direct clinical care, providing peer support or providing linkage services to YPLH. Such a provider was then asked whether or not they had ever interacted with a YPLH with unhealthy drinking. For this purpose, unhealthy drinking was defined as a young person drinking 4 or more, and 5 or more drinks by females and males respectively on at least one occasion in the past month. She shared the list of selected care providers and their telephone contact numbers with the study team, who then purposively approached the care providers during the adolescent clinic days (every Thursday) or called them on phone to inform them about the study and invite them to a face-to-face interview.

All the providers we approached agreed to participate in the interviews and none dropped out midway. The interviews were conducted in a private room provided by the hospital that had been pretested for suitability to record interviews. There were no interruptions during the entire interview process. The Principal investigator (PI) and the research assistant (RA) separately conducted the interviews. The male PI interviewed 3 while the female RA interviewed 7 participants. The interviews were gender concordant. There was no previously established relationship between the PI and the HCPs but the RA was a familiar face at the clinic as she had participated in two previous studies at the same clinic over the previous five years. No one else was present during the interviews except the interviewer and the provider. The participants comprised adult clinic staff, both male and female, whose roles were clinical (doctors, clinicians, nurses), counseling, peer support and linkage facilitation.

A trained female research assistant (NS), with masters in social works and ten years of qualitative research experience, together with a male principal investigator (RFO), who is an addiction psychiatrist and a PhD candidate, with nearly fifteen years of experience as a clinician in mental health, conducted the interviews. The two researchers (NS and ORF) recruited HCPs and obtained written informed consent for both, participation in the in-depth interview (IDI) and for tape-recording the interview, before each interview. The in-depth interviews were face-to-face, audio-recorded and typically lasted about 40 min but varied between 35 and 70 min. All the interviews were conducted in English, by the preference of the HCPs, although the option to interview in the local dialect of Runyankole was available.

The main topics discussed are shown in Table 1 below. The interviewer took notes. No repeat interviews were conducted but interviewers asked key questions twice to see if there was change in perspectives. Interviews were tape-recorded. We provided feedback and maintained our interviewer roles throughout. We tracked and managed time during the interview. Transcripts were not shared with participants for their comments or revisions. A sum of 50,000 Ugandan Shillings (about $ 15) was reimbursed as time and transport compensation to each participant.

**Table 1:** Semi-structured interview topic guide for Health Care Providers

Topic	Sample question
Provider views on alcohol use among YPLH	How is drinking of alcohol an issue among young people living with HIV attending your clinic?
Drivers of alcohol use among YPLH in Mbarara	Why are young people drinking alcohol?
HIV Care Provider Knowledge in and experience with YPLH who drink alcohol	In your role as a health care provider, what do you do to address alcohol use among YPLH? (Probe for the different ways or support that the Health Care Provider offers specifically).
Care capacity for alcohol use among YPLH	What is your capacity to identify clients who use alcohol and provide therapy for them within the HIV clinic? (Capacity to identify, counsel, advise and refer? Follow up mechanisms for clients with alcohol use? )
Health Care Provider Motivation to support YPLH who drink alcohol	Generally, how do you feel about working with young people living with HIV who also drink alcohol? (Probe for what drives the health care provider - skill, passion, experiences, obligation? )

We used a semi-structured interview guide to allow new viewpoints to emerge freely. Interview guides were constructed by the PI, in consultation with the Head of the ISS clinic at MRRH (WRM), and the research assistant (NS) who conducted the interviews with the PI. The guide was informed by prior research and clinical experience. After developing interview questions, we conducted three pilot interviews with HIV care providers at a different clinic i.e., the ISS clinic at Mulago National Referral Hospital in Kampala. We used these pilot interviews only to help refine our guide by ensuring that, the participants understood the questions and their essence, before using it with the providers in our study. We did not derive any themes from this pilot for the purpose of deductive analysis. We also developed a brief socio-demographic data sheet and used it to collect participants’ demographic information like age, sex, education status, and position held/role at the clinic. Interviews were conducted until thematic saturation.

### Ethical considerations

This study was granted ethical clearance by the Makerere University School of Medicine Research and Ethics Committee (Mak-SOMREC-2021-168) and the Uganda National Council of Science and Technology (UNCST-HS2001ES). After explaining the objectives and procedures of the study, written informed consent was obtained from all participants for participation, tape-recording and taking written notes during the interviews. Participants were assured of confidentiality before starting each interview.

### Data management and analysis

The interviews were audio-recorded and later transcribed verbatim by listening and re-listening to each tape by the research assistant (NS). Data coding and analysis were done using the Atlas.Ti computer software for windows [25]. ORF and TA read all the data scripts and developed a data analysis plan based on the objective of the study. This was done in close consultation with the project team. Thematic analysis was used. To find thematic statements, the transcripts were repeatedly reviewed. This way, the two researchers were able to find meaning and understanding of the themes. Thematic statements or phrases were identified and isolated if they stood out as those that captured the essence of the providers’ experiences and perceptions regarding caring for YPLH with unhealthy alcohol use (van Manen 1990). Using the selected themes, the researchers looked for connections across the emergent themes and drew together the emergent themes to produce the most important aspects of HCP’s account. This was done for each participant but in the end, the researchers looked for patterns across all the participants (Smith et al. 2009). Any discrepancy in coding was discussed and consensus reached. After completion of the initial coding, themes and sub-themes were identified, checked and refined in consultation with two senior qualitative researchers (WWM and ARK). Memos describing the patterns and variations in the different segments of retrieved data were written. Writing and re-writing the themes enabled us to develop the interpretations. Verbatim quotations from the data are used to highlight key study findings.

### Study quality assurance

Throughout this study, we promoted trustworthiness by ensuring credibility, dependability, and transferability. For example, to interpret the experience from the viewpoint of the participants, we described the phenomena of meaning from the patients as they were presented to us. We checked and rechecked the written observations we made during the interviews and measured them, as well as our thoughts and observations from the reflective journal, against the participant stories at every phase in the analysis. We were careful to ensure that we deeply reflected on each interview to ensure that we identified only participants’ meanings and not ours. Meeting regularly as a team to discuss certain interviews also helped us reach congruent interpretations. Using a reflective journal, we documented every step of the research and analysis process. We faithfully described the HIV care providers’ perceptions, experience and meanings, which can set a foundation for future research that seeks meaning in the healthcare provider population. Lastly, we reviewed our results with some clinicians in the clinic as well as the head of the ISS clinic of Mbarara Regional Referral Hospital (WRM).

## Results

### Participants’ characteristics

The 10 study participants were HIV care providers working at the Immunosuppression (ISS) clinic of Mbarara Regional Referral Hospital. The majority (70%) of participants were female; their age ranged from 24 to 42 years. One participant declined to state his age but appeared to be in his mid-50s. Participants comprised of Linkage facilitators (*n* = 2), counselors (*n* = 2), peers (*n* = 1), clinical officers (*n* = 2), medical officers (*n* = 2) and medical specialist (*n* = 1) (Table 2).

**Table 2:** Participant characteristics

No.	Sex	Age	Level of education	Role
P1	F	24	High school (6 years)	Linkage Facilitator
P2	F	24	High school (5 years)	Linkage Facilitator
P3	F	42	High school (1 year)	Counselor
P4	F	32	High school (3 years)	Counselor
P5	F	27	Tertiary (Diploma)	Clinical Officer
P6	M	24	Primary school	Peer counselor
P7	F	27	Tertiary (Degree)	Medical Officer (MO)
P8	F	28	Tertiary (Degree)	Medical Officer (MO)
P9	M	40	Tertiary (Diploma)	Clinical Officer
P10	M	Not stated (appears to be in his 50s)	Tertiary (Degrees)	Pediatrician

### Overview

Our results highlighted the perceptions and experiences of caring for YPLH with unhealthy alcohol use among HIV care providers (HCPs) in Southwestern Uganda. Overall, HCP’s accounts of their experiences and perceptions grouped around five main interconnected themes. These were; (1) Intention to care; (2) Provider Attitude; (3) Subjective norms; (4) Perceived Control; and (5) Barriers to care. A summary of themes and subthemes is provided in Appendix 1.

## Theme 1: intentions to care

### Subtheme I: passion for work

The participants in the study generally expressed intentions to care for YPLH. They enjoyed their work and were highly motivated to work with young people living with HIV. Some providers reported that they were just passionate about and derived intrinsic satisfaction from helping young people. Providers were also motivated by the joy they felt when their interventions impacted young people positively as well as the testimonies of change by the young people. These sentiments were espoused by the whole spectrum of providers from linkage facilitators to medical officers, as captured in the excerpts below:

*“…so regardless of which category they fall*,* I just enjoy it and I always try to give them my best. This is because I generally love working with young people; not just those who are HIV positive or use alcohol“***(P7_27-year-old Female Medical Officer)**.

*”It is fun… and the mere fact that you are helping someone to be a better person gives you joy… It gives me peace knowing that I am helping them“***(P1_24-year-old Linkage facilitator).**

*“I always feel so good when I see these fellow adolescents prospering… So I always feel happy when I see that I am restoring hope“***(P6_24-year-old Peer counselor).**

### Subtheme II: concern for Young people

Some providers felt concern about the young people’s future and that of the nation, and this motivated them to care for the young people. This concern drove the providers to help young people achieve their full potential, support them to handle stigma of living with HIV, and restore hope in them. The providers saw the young people as the future of the nation who deserve to be supported to be able to realize their full potential. In addition, the providers were encouraged and inspired to work with young people when they witnessed positive changes in the lives of the young people such as, observing weight gain and viral load suppression following ART, as well as changes in their social life like disclosing HIV status to a partner, marriage proposals and successful completion of school, as observed by this linkage facilitator; *“Some invite us to those functions (e.g. graduation)…. So you really feel well“***(P1_24-year-old Linkage facilitator).**

### Subtheme III: “…no one out there does it.”

Another source of inspiration for the providers to work with young people was the unique characteristics of young people. The providers believed that working with young people living with HIV, particularly addressing their psychosocial issues, was a privilege that should otherwise be provided by parents and schools but it was not. There was a sense that young people’s psychosocial needs were largely unmet by their duty bearers, necessitating HIV care providers to go over and beyond their health mandate. The following response captures this sentiment regarding young people’s unmet psychosocial needs.

*“We go beyond taking care of HIV; we do a lot of things here because we know what we do here no one out there does it. Schools no longer do what we do here. I know parents are in the storms of looking for money; … marital issues; what they know is giving them food*,* sending them to school and giving them the essentials. The rest that is pertaining to the brain; no one is doing it“***(P4_32-year-old Female Counselor).**

Providers in this study indeed showed high motivation and intention to care for the YPLH including those with unhealthy alcohol use however, their intentions to care seemed to be influenced by other perceptions such as their individual attitudes, the practice norms, power and control, and some provider barriers to care.

## Theme 2: individual attitude

Our data showed that HCPs were aware that young people living with HIV used alcohol and other substances. They were also aware of the impact of alcohol and other substance use on YPLH in their care. Two subthemes emerged.

### Subtheme I: recognized concern

As shown in the voices below, providers recognized that alcohol use was a major concern for YPLH in care. They also recognized the burden of other substances such as; *mairungi* [khat], cocaine, *weed* (cannabis), and *shisha* (flavored tobacco). Providers perceived the known burden of alcohol use by YPLH to be an underestimate of its true burden. This is because the providers believed that YPLH underreported their alcohol use and that they told lies, were secretive, and were reluctant to disclose their substance use behaviors to the providers in the HIV clinic. This perception however was not shared by peer counselors who reported that young people were more likely to open up to them regarding their alcohol use.

*“Recently*,* we created a screening tool… It is the clinicians who are doing the screening. . but they have been surprised; our children take alcohol*,* they use drugs*,* they use mairungi*,* our children take cocaine…“***(P3_42-year-old female counselor).**

*“Yes*,* as much as the alcoholics with HIV are not common… some lie to us when we talk to them (about it)”***(P1_24-year-old Linkage facilitator)**.

*“Yes*,* you see they can open up to us fellow peers; you see we always hang out at our own time. They tell us their secrets but to our service providers sometimes they hide when they are being screened“***(P6_24-year-old Peer counselor)**.

### Subtheme II: impact of Alcohol Use on YPLH

Providers understood and acknowledged that unhealthy alcohol use among YPLH harmed their HIV care. Unhealthy drinking was perceived to be a barrier to ART treatment, adherence, viral non-suppression, and poor functioning. A pediatrician in his fifties captured this sentiment in the voice below;

*“If we identify (alcohol problem)*,* we have a counseling team; they first handle and even as clinicians we talk to them if we identify it as a barrier to accessing care; to taking medicine; to being functional and productive… but sometimes we see the problem is much bigger; it is going to addiction… we refer them to our mental health unit…“***(P10_Male Pediatrician)**.

His use of the phrase “If we identify…” suggests that alcohol screening is not commonplace in the clinic and perhaps is used when HIV outcomes are not satisfactory to the clinicians. Some providers confirmed that the cases of unhealthy alcohol use passed undetected during routine clinic days.

## Theme 3: practice norms

Our findings show that the providers’ practice of caring for YPLH with unhealthy alcohol use was largely influenced by the normative clinic operations which were characterized by heavy workload and time constraints. Consequently, some interventions were offered while others, though potentially effective, were left out.

### Subtheme I: interventions used in care

HIV care providers were aware of interventions that may help YPLH with unhealthy drinking, such as counseling, peer support, and referrals. These interventions were often reserved for when the drinking problem was perceived to be excessive. The providers generally perceived young people with unhealthy drinking to be more difficult to deal with because they were more likely to miss appointments and not adhere to their ART treatment. Currently, counseling and peer support are being provided for YPLH with unhealthy alcohol use. HIV counselors are relied upon to provide counseling to enable young people to understand the possible repercussions of alcohol use regarding their health, medication, and the future. Counseling is supposed to tackle all the issues including alcohol, which are likely to affect the individual’s ability to seek medical care, treatment adherence, and viral load suppression. The counselors sometimes succeed but other times they do not, and they in turn refer the cases to fellow counsellors or social workers.

*“These are the people we have to keep sending to the counselor every time they come in. I think I have had to send to the counselor one young adult who came in drunk… This is also a client that was coming in for intensive adherence counselling (IAC); adherence is already not very good; and viral load“***(P8_28-year-old Medical Doctor)**.

Peer support was another intervention used by the providers. Here, the newly identified young person with unhealthy drinking is attached to a peer, usually older, who previously overcame his own drinking. Peer support is also used to prevent alcohol use among young people through psychosocial peer meetings where alcohol use is talked about and discouraged to prevent it from happening. The peer counselors in our study perceived that YPLH preferred their interventions over those given by the health care workers. At these meetings, it is reported that the issues that drive young people to use alcohol are identified and addressed. The following voices espouse these and other perceptions on peer support:

*“If it is like a teenager*,* we will attach someone who is slightly older*,* like 18 or 20 years who has had a similar history to provide support“***(P8_28-year-old Female Medical Officer).**

*“Psychosocial support*,* peer support*,* is really very important because we have had a few young people who had challenges; they have graduated from that and they are now ok. When we attach these other ones to them; they really have a positive change than when the health workers talk to them“***(P5_27-year-old Female Clinical officer)**.

One respondent however, observed that the meetings did not happen as often as they ought to due to the high client numbers on routine clinic days. *“… and that is something [psychosocial meetings] that is not happening as I really want it to happen. You find it happens like once in a year or it doesn’t happen at all“***(P5_27-year-old Female Clinical officer)**.

Referrals was yet another intervention used to care for YPLH in the clinic. Referrals were made within or outside the clinic. This determination of where to refer was predominantly the work of the linkage facilitators. Internal referrals between the various categories of HIV care providers attached to the adolescent HIV clinic were more common than referrals outside the clinic.

*“We refer them to the counselors… he will be followed up*,* have home visits*,* attach him to other adolescents that don’t take alcohol*,* so that they can help solve their problems… If they find out that he has a psychiatric problem*,* then he will be referred but that’s rare“***(P1_24-year-old Linkage facilitator)**.

Although such referrals included unhealthy drinking, they were not entirely for alcohol use alone. For instance, the linkage facilitators refer clients to the counselors who counsel them, conduct home visits, and organize peer support for the concerned clients. Counselors also referred to fellow counselors if the client was not responding fast enough to HIV care. And if the counselors deemed the client to be so severely sick as to require specialist psychiatric attention regardless of the alcohol-drinking status, they in turn referred to the Department of Psychiatry, a medical department of the hospital or to community rehabilitation centers (in case of alcoholism) but such severe cases were said to be rare.

*“If we identify (alcohol problem)*,* we have a counseling team; they first handle and even as clinicians we talk to them if we identify it as a barrier to accessing car; […] but sometimes we see the problem is much bigger; it is going to addiction… we refer them to our mental health unit…“***(P10_Male Pediatrician)**.

### Subtheme II: known but unutilized interventions

All HCPs reported that they had no formal training in managing alcohol use problems but they were aware that some medications, health education and technological interventions are helpful but they were not used in routine care of young people with unhealthy drinking. Where it was deemed that medications were necessary to manage unhealthy alcohol use among the YPLH, this became ground for referral to the Department of Psychiatry for further management as noted by this pediatrician…*"If we identify (alcohol problem)*,* we have a counseling team; […] but sometimes we see the problem is much bigger; it is going to addiction… we refer them to our mental health unit…“***(P10_Male Pediatrician)**.

Some study participants also reported that health education talks may be useful for YPLH with unhealthy alcohol use if only such talks would directly target alcohol. However, when such health talks occurred at the clinic, they were reportedly focused on issues such as; self-discipline, safe sex, sexual abstinence, family planning, treatment adherence, help-seeking, positive living, the role of ARVs, viral load suppression, HIV sero-status disclosure, sharing personal experiences, and managing ARVs while at school. according to two medical doctors, alcohol use was not talked about nor linked to the poor HIV care outcomes such as viral non-suppression during these health talks.

*“No [talk about alcohol use]*,* basically those group meetings are based on adherence*,* positive living*,* and viral load suppression; yes. Sometimes we overlook what causes the non-suppression“***(P7_27-year-old Female Medical Officer)**.

*”… but alcohol is that one thing that we don’t talk about a lot… Yes*,* I have seen it on the Rota a few times but we don’t usually talk about it a lot… the health education sessions that I have attended*,* it is not something that comes out clearly that we are trying to deal with…“***(P8_28-year-old Female Medical Officer).**

A peer counsellor in the study indicated that technological interventions such as setting up online websites and creating computer applications to address issues of the adolescent alcohol use, may be effective if explored. Online strategies were perceived to be promising since they tended to offer the young people more confidentiality compared to the physical meetings but these are not yet in effect.

### Subtheme III: Heavy workloads and Time constraints

The providers felt that the routine clinic days were so busy that the heavy workloads rendered some otherwise good interventions unworkable. For instance, it was reportedly not possible to organize psychosocial group meetings for the young people due to the large client numbers on the routine clinic days.

*“You find that we can’t conduct psychosocial groups on clinic days because the clinic is very full. And when you call them to come from their homes like during holidays*,* you have to have something (for them) … which sometimes the budget doesn’t allow***(P5_27-year-old Female Clinical officer).**

## Theme 4: power and control over Care

Data showed that the providers, especially the medically trained ones such as medical doctors and clinicians, lacked confidence in their ability to see a YPLH through their alcohol use and that they got easily frustrated and discouraged by the lack of improvement in the young people they handle. Providers also felt disempowered because some of their interventions required collaboration and support from parents and the institution yet these were rarely availed.

### Subtheme I: ineffective interventions

Providers complained that young people often neither followed their guidance on treatment adherence nor on leaving alcohol and drugs leading to development of complications. It appears providers lacked in confidence and patience as they wanted to see immediate change in their clients.

*“But then the challenge comes in when you want to help them and they feel that they don’t want your help… the bad situation that pushes me down is*,* if you tell them to please adhere to the drugs*,* stop using these drugs and they fail and then they present with complications at a later stage”* (**P5_27-year-old Female Clinical officer)**.

*“I rarely get demotivated about my work. But if I find this is the client that I took my 2 hours on; we talked and we really had a plan. It is the client …who decides*,* we are going to do this… You gave me this and now you are doing the opposite… Yes*,* it happens“***(P7_27-year-old Female Medical Officer).**

Notably however, although some providers felt frustrated with caring for their clients, they did not abandon them. In such cases, referrals were made between providers (e.g. counsellors and peers) or to the mental health unit of the hospital.

### Subtheme II: support from families and Hospital Management

Providers were motivated to care for YPLH with unhealthy alcohol use but they perceived that they had no full authority and control over their interventions. Some interventions were perceived to be effective but could not be delivered by the provider without the support from the YPLH’s family or without financial support from the institution. It was perceived that the young people’s families as well as the institution were at times unable or unwilling to provide the necessary support for the young people, especially the costly interventions.

*“So the de-motivators are not because of them (young people); sometimes it is the parents at home… You can discuss something with them and when they go home*,* they do the opposite. Or you request for something to help the young people and they don’t provide“***(P4_32-year-old Female Counselor).**

*“…you just feel maybe the system is not supporting you to do what you would like to do for them. But it is not their problem as young people…“***(P10_Male Pediatrician).**

*“The de-motivators are not part of my adolescents; they are beyond; like they are administrative*,* you ask for something and you don’t get it“***(P4_32-year-old Female Counselor).**

*“Yes*,* and lack of resources to have them helped the way I feel would help them better. That demotivates me a bit. Even when you present your idea that we would like to do this and that for the adolescents; but it can’t go anywhere without funds…“***(P5_27-year-old Female Clinical officer).**

## Theme 5: barriers to Care

HIV care providers reported on several barriers to caring for young people living with HIV with unhealthy alcohol use. These barriers seem to impact on all above themes especially the intention to care.

### Subtheme I: knowledge and skills gaps

Providers lacked knowledge and skills on how to intervene correctly with YPLH with unhealthy drinking. As shown in the counselor voice below, there was a sense of helplessness about how to handle YPLH’s problems such as unhealthy drinking.

*“By the way*,* we were never trained to deal with adolescents*,* when we got here; they just said that you will work here. I think it is by the grace of God*,* someone finishes going through the clinic…“***(P4_32-year-old Female Counselor).**

Even the more qualified providers such as medical officers decried the lack of or low levels of knowledge in caring for YPLH with unhealthy alcohol use. Most of what they do to try to help YPLH was learnt on the job or through anecdotal information gathered during other trainings unrelated to alcohol or substance use. Thus, medical doctors relied on the basic knowledge on substance use disorders they gained from the university during medical training but that this training was general, brief, and not necessarily about young people living with HIV. A medical doctor thus noted:

*“I just have an idea (on SUD counselling and psychotherapy) but to be very honest*,* I have not had any training*,* I have just had bits and pieces that I have had in different counseling sessions*,* in different presentations. But I have not had any training***(P8_28-year-old Female Medical Officer).**

None of the other non-medical providers such as counselors, peers, and linkage providers had ever undergone specific training in alcohol use or other substance use disorders, especially among young people living with HIV. These providers expressed a need for new and refresher (continuing) training to equip them with knowledge and skills in handling young people living with HIV especially those using alcohol and other addictive substances as well as dealing with newly emerging challenges. Some specifically expressed the need for training in psychotherapy especially for young people.

*“I have attended a few trainings but about handling people who use alcohol*,* I don’t have training; I wish I could get“***(P6_24-year-old Peer counselor).**

*“Like specifically in alcoholism in adolescents and young people…. If they can even take us in the rehabilitation centers and we see what these people are doing there*,* we can learn a lot.“***(P4_32-year-old Female Counselor).**

### Subtheme II: remuneration and incentives

Low or delayed payments of salaries was perceived to be a major barrier to care for YPLH. The lower cadre providers such as the linkage facilitators and the counselors felt that when it came to unhealthy drinking, they did the bulk of the interventions and yet their pay was not commensurate with the workload.

*“Sometimes you don’t have much and you can’t have lunch as they take long to pay. You have to talk to adolescents; you are hungry*,* thirsty and tired so money is needed“***(P1_24-year-old Female Linkage facilitator).**

*“There are some of those that feel like it’s enough for them. I’ve studied*,* I have graduated*,* I have different ambitions*,* and I have different qualifications besides my degree…; so I feel I’m entitled to something bigger… if I could get something bigger because my diploma still connects me to young ones so I’d go for that“***(P2_24-year-old Female Linkage facilitator).**

## Discussion

In this study, we sought to explore the perceptions and experiences of HIV care providers (HCPs) towards caring for young people living with HIV (YPLH) with unhealthy alcohol use in Southwestern Uganda. Overall, the perceptions of the HCPs towards caring for YPLH with unhealthy alcohol use can be attributed to individual attitudes, practice norms, intentions, and perceived control. When engaging with YPLH, providers held some enduring attitudes, clinic specific practice norms, expressed intentions to care, and perceived power and control over such care.

We found four key results. Firstly, HCPs’ attitudes were that unhealthy drinking among YPLH was a serious health concern that impacts their HIV treatment. Secondly, providers’ subjective norms as people working with physical health issues of young people meant that they knew of some effective interventions for unhealthy drinking but did not apply them, in part due to their normally busy clinic days and the power dynamics in play at the clinic. Thirdly, HCPs’ intentions to care for YPLH with unhealthy alcohol use was motivated by the passion for their work as well as their concern for young people generally, however, such intentions were barred by lack of adequate knowledge and skills and low support from families and hospital management. Lastly, HCPs’ perceived control over caring for YPLH with unhealthy alcohol use was influenced by their lack of confidence in the results of their interventions which sometimes resulted in a lack of progress, and they lacked control to institute interventions of their choice due to administrative red tape. Our findings thus conform to Azjen and Driver’s Theory of Reasoned Action (previously theory of planned behavior) [26, 27].

The data from HCPs in our study show that they have intentions of caring for YPLH with unhealthy alcohol use attending the adolescent clinic. This care intention is exemplified by the perceived passion for their work and the general concern most of them have for young people whom they perceive to be the ‘future of the nation’. The HCPs’ intention to care for YPLH however is barred by a lack of knowledge and skills, and low incentives and poor remunerations. Providers reported low skills and knowledge to be able to identify and intervene in alcohol use problems of young people. None of the HIV care providers at the adolescent clinic had had any formal training in handling young people living with HIV who drink alcohol. This training gap is also experienced similarly in primary health care settings elsewhere [28, 29]. The lack of training notwithstanding, the care providers in our study showed much willingness and enthusiasm to receive such trainings and some even suggested a rotation at a rehabilitation center so as to get hands-on experience in handling young people who drink. This finding is consistent with the previously observed need for, as a starting point, training HIV care providers in integration of the evidence based screening and brief intervention (SBI) [30] into their routine care of YPLH. This would entail asking screening questions such that positive screens receive care provider delivered information and motivation-enhancing interviews (MI) [30]. SBI and MI have been found to be effective for and acceptable in HIV populations [31, 32] and may be effective in a setting such as the Mbarara Regional Referral Hospital Adolescent Clinic, where HCPs have intentions to care for YPLH with unhealthy alcohol use.

HCPs’ intention to care for YPLH with unhealthy alcohol use is partly influenced by their individual attitudes towards working with YPLH with unhealthy alcohol use. Our findings, similar to those in other primary care settings [33, 34], show that HIV care providers recognized alcohol use as a serious concern among young people attending their clinic. In fact, efforts where possible were made to screen, make a diagnosis and refer to counselors and peers. However, the providers did not routinely engage in screening and briefly intervening for YPLH who drink alcohol despite citing alcohol use as a barrier to HIV care. Although the reasons for such perception are not entirely clear from our data, studies have identified such factors as provider discomfort and avoidance of asking about and discussing alcohol use in primary care as potential barriers to interventions [34, 35]. In our study, only when HIV care is affected, do the care providers assess for alcohol as a possible cause. This finding speaks to a lack of awareness about the true magnitude of unhealthy drinking by YPLH among HCPs in this study. This is in keeping with the general global picture where primary care providers are largely unaware of the magnitude of alcohol use problems among patients seeking care with in their facilities [22, 35, 36] and are hindered to ask about alcohol use because of concerns about patient-provider relationship [37]. These findings call for HIV care provider sensitization and training to identify unhealthy alcohol use among YPLH.

Another influence on HCPs’ intention to care for YPLH with unhealthy drinking was subjective practice norms. In the adolescent clinic, things are done a certain way by specific people working there. The who-does-what-when is clear. Our findings highlight that, where unhealthy alcohol use by YPLH has been identified, HCPs know and employ two main interventions i.e., counseling and peer support to effect behavior change. The counseling role was reserved for HIV counselors, while the peer support role for a peer supporter who preferably has a lived experience with unhealthy drinking and HIV. Studies have shown that even briefly engaging patients in a discussion about their alcohol intake greatly decreases alcohol use among people living with HIV [31, 38]. Studies have also shown that peer-led activities may prevent youth alcohol use [39] through peer helping [40] and influencing adolescent social perceptions of alcohol use [41]. Other benefits of peer support include reduced alcohol use and alcohol use disorder relapse rates, improved relationships with treatment providers and social supports, increased treatment retention, and greater treatment satisfaction [42]. This lends credence to both the counseling and peer support that are provided at the adolescent clinic. However, due to the clinic norms, it was apparent that no other providers such as clinicians and medical doctors ever engaged with clients in this regard. This is partly because they perceive their role in the care of YPLH to be limited to HIV care only. So the clinic majorly relied on linkage providers for making referrals and HIV counselors and peers to provide counseling for YPLH with unhealthy alcohol use. All other HCPs therefore refer to these categories of providers. Moreover, the peers and counselors themselves have had no training in providing help to YPLH with unhealthy drinking. However, given the normative and resource constraints in the clinic, with proper training, peers and counselors can be a great resource for task-shifting alcohol use screening and interventions for YPLH in such primary care settings [34, 35].

Another important perception that largely informs HCPs’ intention to care for YPLH with unhealthy alcohol use is their perceived power and control over the care they provide. From our findings, HCPs were frustrated by the failure to see any significant improvements in the young people despite their best efforts to treat them. Some providers had stigmatizing attitudes towards YPLH and unhealthy alcohol, and blamed YPLH for not listening to their advice and for drinking excessively. They perceived that the young people’s families were disengaged when it came to collaborative care. They also perceived that the institution did not support their efforts towards caring for the young people, especially financially. The care providers wished to work with families of young people and the institutional administration collaboratively. However, as things stand, HCPs lack perceived authority and control to institute effective interventions unless the institution clears them to do so. According to the TRA, the lack of things that will make it easier to perform a behavior affect perceived behavioral control [27]. So the HCPs in our sample are rather disempowered to perform the task of caring for YPLH with unhealthy drinking. Yet, HCPs, like most primary care providers [34], are potentially a great resource for identifying and caring for YPLH with unhealthy alcohol use in Southwestern Uganda when targeted for trainings and interventions for YPLH with unhealthy alcohol use. Moreover, their motivation, passion and concern for young people can be leveraged for better care of YPLH with unhealthy alcohol use. Future studies are needed to focus on understanding the family-healthcare dynamics and how to create an enabling cooperation that supports recovery of YPLH with unhealthy drinking.

### Strengths and weaknesses

Among the study’s limitations is the likely social desirability bias which may have influenced responders’ apparent underreporting of alcohol use interventions among young people. Although we purposively chose HCPs who had reported working with young people living with HIV with unhealthy drinking, fear of exposing a non-standardized intervention technique or failure to identify unhealthy drinking, may have resulted in keeping silent about it. We did not include a measure to assess for this social desirability effect. Another limitation is that we only report the subjective accounts of HIV care providers at a large urban HIV clinic in Southwestern Uganda but not the accounts of family members whose perspectives of caring and living with YPLH with unhealthy drinking is crucial. People living with YPLH with unhealthy drinking often try various interventions available in their communities. Such interventions often run alongside clinic interventions. There is no study that explores this mutual care of YPLH with unhealthy drinking. Further studies could focus on this collaborative care. Lastly, the qualitative nature of the study limits generalizability.

Our study has some major strengths; firstly, we are not aware that any such study has previously been done in our setting. Secondly, being a qualitative study, it provides deep insights into perceptions of care providers in the clinic which can be valuable in the strengthening the program.

## Conclusions

HIV care providers are important stakeholders in the care of YPLH. Our findings suggest that HCP’s intention to care for YPLH with unhealthy alcohol use is influenced by their attitudes which are characterized by motivation, passion and concern for YPLH. They recognize unhealthy alcohol use among them as a major health concern that impacts HIV care. Intentions to care are also influenced by the subjective norms at the adolescent clinic that sees more senior clinicians relegating the responsibility of caring for YPLH with unhealthy alcohol use to counselors, peers and linkage facilitators. As such, some would-be effective interventions though known to the providers, are not being applied. HCPs are also disempowered to help YPLH with unhealthy alcohol use due to perceived lack of authority and control, as well as lack of knowledge and skills. Further research that seeks to develop and build capacity of HCPs to provide simple HCP-delivered interventions for YPLH are needed. Delivering such interventions will require addressing provider attitudes, subjective norms and perceived control.

### Electronic supplementary material

Below is the link to the electronic supplementary material.

Supplementary Material 1

## Data Availability

The data generated and analyzed in this study are not publicly available so as not to compromise individual privacy and anonymity.

## Abbreviations

PWH: Persons with HIV
YPLH: Young people living with HIV
HCPs: HIV care providers
